# Knowledge, stigma, health seeking behaviour and its determinants among patients with post kalaazar dermal leishmaniasis, Bihar, India

**DOI:** 10.1371/journal.pone.0203407

**Published:** 2018-09-07

**Authors:** Pavan Garapati, Biplab Pal, Niyamat Ali Siddiqui, Sanjiva Bimal, Pradeep Das, Krishna Murti, Krishna Pandey

**Affiliations:** 1 Department of Pharmacy Practice, National Institute of Pharmaceutical Education and Research, Hajipur, Bihar, India; 2 Department of Biostatistics, Rajendra Memorial Research Institute of Medical Sciences (Indian Council of Medical Research), Agamkuan, Patna, Bihar, India; 3 Department of Immunology, Rajendra Memorial Research Institute of Medical Sciences (Indian Council of Medical Research), Agamkuan, Patna, Bihar, India; 4 Department of Molecular Biology, Rajendra Memorial Research Institute of Medical Sciences (Indian Council of Medical Research), Agamkuan, Patna, Bihar, India; 5 Department of Clinical Medicine, Rajendra Memorial Research Institute of Medical Sciences (Indian Council of Medical Research), Agamkuan, Patna, Bihar, India; Universita degli Studi di Parma, ITALY

## Abstract

**Background:**

Lesishmaniasis is a neglected tropical disease endemic in Bihar, India. Inappropriate health seeking behaviour of post kala-azar dermal leishmaniasis (PKDL) patients may increase the disease duration, severity and transmissibility. Simultaneously, lack of knowledge and perceived stigma may also increase the length of delay in receiving treatment. This ultimately effects the kala-azar elimination program.

**Methods:**

A cross sectional study was conducted in 120 confirmed PKDL patients, aged 18 years and older. Data related to knowledge and health seeking behaviour was collected by a pre-tested questionnaire. EMIC stigma scale was used for assessing the perceived stigma. Patients were personally interviewed after taking informed consent. Data analysis was done by using SPSS 16 software.

**Results:**

The time between appearance of symptoms and first medical consultation (patient delay) ranged from 15 days to 5475 days (15 years) with a median of 285 days. The time between first medical consultations to onset of specific treatment (system delay) ranged from 2 to 5475 days with a median of 365 days. Many patients approached first to quacks (8.4%), homeopathic and ayurvedic practitioners (25.8%) upon recognition of symptoms. Majority of the patients (68.3%) had poor knowledge about PKDL and its vector. Type of skin lesions and gender had significant association with patient delay and system delay respectively (p<0.05). Distance to primary health centre (PHC) had significant association with patients delay as well as system delay (p<0.05). Patients with younger age, unmarried and polymorphic lesions had higher stigma (p<0.05). Patients with PKDL feel stigmatized in different areas.

**Conclusion:**

PKDL treatment delays were unacceptably high and patients had poor knowledge compounded with feelings of stigmatization. To reduce the delay, a system may be evolved to establish some sort of public-private collaboration, besides awareness programs should be tailored, and implemented for improving the patient education regarding the disease and its linkage with VL.

## Introduction

Leishmaniasis is a vector-borne disease, caused by protozoan parasite genus *Leishmania*. This is one of the neglected tropical diseases affecting the poorest of the poor in developing countries, who are at the risk of over 350 million people[1]. Clinical manifestations of the disease ranging from self-curing cutaneous leishmaniasis (CL) to severe visceral leishmaniasis(VL) leads to death if left untreated. Globally 90% of VL cases occur in India, Bangladesh, Nepal, Brazil, Ethiopia and Sudan[2]. Post kala-azar dermal leishmaniasis (PKDL) is a dermatological consequence of VL, reported in endemic areas of *Leishmania donovani*. The disease presents with different types of skin lesions likely hypopigmented macule, erythematous papule, nodule and mixed polymorphic forms of the lesions. PKDL develops in 5–10% of the treated VL cases within 2–3 years in India [3].

Indian government has planned to eliminate VL and PKDL by 2020. Community participation as well as cooperation from the affected individual is essential for successful control and elimination of any disease. Prevention, control and elimination of any disease mainly depends on the knowledge, attitude and practice of the population regarding the disease simultaneously, poor knowledge may lead to inappropriate health seeking behaviour and treatment delay[4]. It is essential for program managers to understand the level of knowledge, attitude, preventive practice and health seeking behaviour of the patients for effective planning and implementation of the kala-azar(KA) control program. PKDL is not a fatal disease, but patient may serve as a durable reservoir for *anthropometric leishmaniasis* transmission until they get treated [5]. Delay in diagnosis and treatment also increases the risk of transmissibility, morbidity and severity of PKDL. In India, there is no data, presenting knowledge, attitude, practice and health seeking behaviour of PKDL patients and how health professionals perceive the disease so this study may fill the gaps in the data. A study on health seeking behaviours in Bangladesh revealed that the majority of the PKDL inflicted patients are not having knowledge about the disease and a considerable number of patients initially approach pharmacy shop, homeopathic and ayurvedic practitioners leading to delay in diagnosis and treatment[6]. Study on VL in Ethiopia reported that people are not having good knowledge regarding disease transmission, signs and symptoms and the infectious origin of the disease[7]. Similarly, a survey of knowledge, attitude and practice related to cutaneous leishmaniasis and sand flies in Punjab, Pakistan revealed the lack of knowledge among communities related to disease and vector management practices[8]. Bihar, which is an endemic zone for leishmaniasis, revealed poor knowledge about symptoms, infectious nature, mode of transmission and preventive measures of the VL[4].

Stigma is an another important factor which leads to social and psychological burden due to impaired quality of life, social exclusion and poor mental health[9]. Moreover, it interferes with the therapeutic outcome of the disease through its effects on treatment seeking behaviour and drug compliance[10]. Social stigma in PKDL patients is hypothesized to be more when the skin lesions are present on exposed body parts[11]. However, to the best of our knowledge, no study has reported the burden of stigma in PKDL patients, despite high endemicity of the disease in India and many other parts of the world. With the view of the above, we therefore, planned to assess the knowledge, stigma due to PKDL, health seeking behaviour, and to identify the influencing factors among PKDL patients.

## Materials and methods

### Study area and setting

Rajendra Memorial Research Institute of Medical Sciences (RMRIMS), Patna, Bihar is a tertiary level public health facility, which has specifically indoor facilities for VL and PKDL case management. It is one of the known referral center which delivers VL & PKDL services free of cost in Bihar. This was hospital based across-sectional study conducted from July 2017 to March 2018 at RMRIMS, Patna, Bihar, India. Patients admitted and diagnosed as confirmed PKDL case and meeting the criteria of study participant were selected successively and interviewed.

### Study participants

Confirmed PKDL patients of both genders, aged between 18 to 70 years were the study participants. The diagnosis of PKDL was done by rK39 test followed by histopathological examination of leishmanial amastigote through a slit skin smear. Patients with skin lesions other than PKDL and having any disability or concomitant illness were excluded. Subjects who did not respond to all the questions or who left before completion of interview were also excluded. Study participants were informed about the aims and objectives of the study prior to starting the interview. Interview was conducted in local language i.e. “Hindi”. Study participants were recruited, following a written informed consent, until the required sample was reached (120 PKDL cases).

### Sample size

The sample size was calculated using Open Epi software **Version 3.01**. Prior to actual sample size calculation, a pilot study was performed among 20 PKDL patients to assess the feasibility and sample size of the study. Based on the result of this pilot study proportion of patients having accurate knowledge about disease, positive attitude and preventive practice were 4%, 10% and 26% respectively at confidence interval 95%, design effects 1%, the required sample size was found to be 46, 83 and 120 respectively. Therefore, considering largest sample, 120 PKDL patients were included in our study. Data of these 20 patients were excluded from actual sample size.

### Variables and data sources

The main outcome variables in health seeking behavior were median patient delay, health system delay and total delays (in days). Patient delay was defined as the time between the appearance of lesions recognized by the patient and first approached to any health care provider. Health system delay was the time between approach to any health care provider, and the onset of specific treatment. Total delay was the time between origin of symptoms to commencement of definite treatment.

Twelve items stigma scale was used for assessing the perceived stigma. This scale was adopted from the explanatory model interview catalogue (EMIC) stigma scale. It is a semi structured scale used in many other neglected tropical diseases including skin diseases like onchocercal skin disease[12], buruli ulcer[13], leprosy [14,15], tuberculosis [16,17] and many other psychological disorders [18,19] for assessing perceived stigma. This scale contains 12 items and each question has four options ‘no’ ‘uncertain’ ‘possibly’ ‘yes’ all questions are scored from 0 to 3, maximum obtainable score is 36 and minimum is 0. Higher the score of EMIC higher the stigma.

### Data collection and data quality assurance

The pretested semi structured questionnaires were used to collect information from the participants. At the end of the interview, health education was given to the patients with the intention of reducing stigma towards PKDL.

Questionnaires were administered by a trained interviewer. The training of interviewer and supervisors emphasized issues such as data collection instruments, inclusion–exclusion criteria, and record keeping. The principal investigator and supervisors coordinated the interview process, spot-checked and reviewed the completed questionnaires on a daily basis to ensure the completeness and consistency of the data collected. They also conducted random quality checks by reinterviewing about 10% of the respondents. Interviews were conducted by visiting every patient at their bed site in the indoor ward of RMRIMS, Patna. Questionnaires consisted of four sections:1) socio-demographic characteristics of the participants such as age, gender, marital status, education, occupation, income, etc. 2) Knowledge, attitude towards disease, and vector and practice to control the disease 3) health seeking behaviour and 4) perceived stigma.

Slit skin smear positive for LD bodies, HIV, hepatitis B and C status, etc. were verified from the individual patient treatment records available in indoor ward of RMRIMS, Patna.

### Data analysis

Data analysis was done with the help of SPSS version 16. Descriptive statistics such as frequency, percentage, mean, median and standard deviation were used to describe clinico-demographic parameters and knowledge, attitude, practices (KAP’s) of PKDL patients. Mann-Whitney U test was used for comparison of medians between two groups and the Kruskal-Wallis H test for comparison of medians in more than two groups. Multiple linear regression models were used to identify the most important determinants of treatment seeking behaviour of PKDL patients.

### Ethics statement

Ethical approval was taken from the Institutional ethical committee of RMRIMS (24/RMRI/EC/2017). Written informed consent was taken from all the eligible patients. Informed consent was presented in Hindi language. After completion of interview session clinical signs and symptoms, vector, transmissibility and preventive measures of PKDL were explained to the study participants. Patients were also ensured about the anonymity and confidentiality of data.

## Results

### Clinico-demographic and health seeking behaviour

A total of 124 patients were interviewed and 4 patients were excluded from the study due to incomplete data. Mean age of the patients were 30 years, ranged from (18 to 70 years). Patients consisted of 63.3% males and 36.7% females. Patient delay ranged from 15 to 5475 days with a median of 285 days (mean: 585, IQR: 120 to 730 days). Within 1 year, 71% of patients were approached to health care provider after developing skin lesions. Illiterate or patients educated up to primary school and laborer reported equal delay of 365 days. No significant difference observed in patient delay with respect to gender (p = 0.31), age groups (p = 0.21), residence (p = 0.83), marital status (p = 0.69), lesion location (p = 0.12) and type of skin lesions (p = 0.68). Health system delay ranged from 2 to 5475 days with a median of 365 days (mean: 802, IQR: 60 to 1460 days). Significant differences in health system delay was observed with the choices of first health care provider (p = 0.00) and distance to primary health care center (p = 0.01). Total delay ranged from 37 to 9125 days (25 years) with a median of 730 days. Total delay was significantly different (p<0.05) with regards to the occupation of the patients, choices of first health care provider and distance to PHC. The details clinico-demographic characteristics and different types of delay are presented in **Table 1**.

**Table 1:** Clinico-demographic characteristics and its relation to health seeking behaviour of PKDL patients (n = 120).

Characteristics	n (%)	Patient delay		HS delay		Total delay	
		Median(IQR)	p value	Median(IQR)	p value	Median(IQR)	p value
**Gender**							
Male	76(63.3)	365(610)	0.313	557(1362)	0.071	1095(1890)	0.089
Female	44(36.7)	225(238)		180(1046)		452(1576)	
**Age**							
18–30	75(62.5)	240(480)	0.214	365(1400)	0.439	730(1608)	0.340
31–50	41(34.2)	365(550)		365(960)		1095(1920)	
>51	4(3.3)	120(1118)		139(1705)		257(2731)	
**Residence**							
Urban	11(9.2)	365(1400)	0.830	915(2740)	0.133	2190(2650)	0.131
Rural	109(90.8)	270(530)		365(1218)		730(1574)	
**Marital status**							
Married	78(65)	240(580)	0.699	270(1400)	0.439	730(1920)	0.750
Unmarried	42(35)	365(625)		365(1158)		730(1600)	
**Education**							
Illiterate	40(33.3)	365(550)	0.017	467(1325)	0.440	1322(2100)	0.119
Primary school	17(14.2)	365(322)		545(1050)		730(2028)	
Secondary school	32(26.7)	255(562)		302(1331)		648(1859)	
Intermediate	21(17.5)	120(328)		150(1035)		360(1462)	
Graduate/more	10(8.3)	135(404)		547(2297)		775(2837)	
**Occupation**							
Farmer	14(11.7)	195(558)	0.014	1277(2185)	0.063	1626(2709)	0.012
House wife	28(23.3)	240(185)		180(346)		452(1298)	
Business	8(6.7)	75(585)		210(119)		330(1828)	
Student	13(10.8)	120(488)		240(1035)		360(1858)	
Labor	39(32.5)	365(1280)		730(1280)		1460(2195)	
Unemployed	11(9.2)	240(215)		150(610)		515(855)	
Job	7(5.8)	60(335)		180(1065)		545(1048)	
**1**^**st**^ **health care provider**							
Quacks	10(8.4)	287(208)	0.004	1550(1475)	0.000	1822(2041)	0.000
PHC	33(27.5)	180(180)		20(53)		210(248)	
H & A	31(25.8)	365(915)		1460(1095)		2190(1825)	
Private practitioner (Allopathy)	46(38.3)	365(558)		365(922)		730(1465)	
**Lesions location**							
Exposed parts	31(25.8)	180(275)	0.126	150(1035)	0.068	360(1585)	0.075
Unexposed parts	2(1.7)	1140		2920		4060	
Both	87(72.5)	365(580)		365(1370)		730(1890)	
**Type of lesions**							
Monomorphic	56(46.7)	302(342)	0.688	302(1400)	0.937	730(2083)	0.662
Polymorphic	64(53.3)	285(632)		365(1005)		730(1525)	
**Distance to PHC**							
**<**5 km	43(35.8)	210(275)	0.005	180(1050)	0.010	607(1340)	0.002
6–17 km	56(46.7)	240(215)		365(1392)		710(1525)	
>18 km	21(17.5)	730(1370)		1095(1918)		2190(2205)	

PHC: Primary health center, H & A: Homeopathic and Ayurvedic, HS: Health system.

km: kilometre.

As regard to knowledge about PKDL, good proportion (72.5%) of the participants were familiar with kala-azar however, in case of PKDL it was much lower (31.7%). Regarding signs and symptoms of PKDL, majority (62.5%) of the respondents were not aware and 42.5% did not know about the vector which transmits the disease. Most of the patients (67.5%) were not having knowledge about breeding places and biting time (75%) of sand fly. Details about the knowledge on PKDL are presented in **Table 2**.

**Table 2:** Knowledge about PKDL among PKDL patients (n = 120).

Characteristics	Categories	n (%)
Heard of kala-azar	Yes	87(72.5)
	No	33(27.5)
Heard of PKDL	Yes	38(31.7)
	No	82(68.3)
Symptoms of PKDL	Stomach ache	1(0.8)
	Skin lesion	42(35)
	Fever	2(1.7)
	I don’t know	75(62.5)
Vector for leishmaniasis	Sand fly	39(32.5)
	House fly	1(0.8)
	Mosquito	29(24.2)
	I don’t know	51(42.5)
Can you identify sand fly	Yes	17(14.2)
	No	103(85.8)
Breeding places of sand fly	Moist places	3(2.5)
	Cow dung	12(10)
	Fresh water	1(0.8)
	Unhygienic conditions	20(16.7)
	Soil	3(2.5)
	I don’t know	81(67.5)
Biting time of sand fly	Dusk & dawn	3(2.5)
	Midnight	17(14.1)
	Day time	8(6.7)
	At any time	2(1.7)
	I don’t know	90(75)
Know the nearest health facility	Yes	118(98.3)
	No	2(1.7)

n = total number of patients.

Majority of the patients (78%) reflected positive attitude and were of the view that PKDL is a curable disease. About 80% of the participants responded that PHC’s are not equipped to manage PKDL cases at the facility. The majority of the patients were using bed net while sleeping (85%) and half of the participants (50%) had sleeping habit outdoor. It is surprising to note that good proportion (40.8%) showed positive attitude towards the control of sand fly through insecticidal spray (DDT/synthetic pyrethroids). Doctors were the main source of information (65%) about the disease.

Most affected area of stigmatization (68.3%) was shame and embarrassment experienced by the PKDL patients, followed by decision to stay away from social group (46.7%). It is worrying to observe here that sizable proportion (15.8%) keeps their disease status confidential. One of the vital drawbacks of stigmatization noticed in this study was difficulty in arranging marriage (24.2%) amongst affected unmarried individual. Details result of stigmatization was described in **Table 3**.

**Table 3:** Mean stigma score for items of stigma scale and percentage of positive responses.

Stigma questions	Mean score	% saying yes	n
Keep others from knowing	0.6	15.8	19
Think less of yourself	1.85	38.3	46
Embarrassed or shamed	2.60	68.3	82
Receive less respect from other	0.78	5.8	7
Adverse effect on others	0.57	8.3	10
Others avoided you	0.42	3.3	4
Refuse to visit your home	0.21	0.8	1
Others think less of your family	0.34	3.3	4
Difficult to marry(if unmarried)	0.86	24.2	29
Problems in marriage (if married)	0.24	0.8	1
Asked to stay away from work	0.58	0.8	1
Decided to stay away from social groups	2.13	46.7	56

n = number of patients responded yes.

Median stigma score was found to be 11 ranging from 2 to 25. Stigma score was not influenced by gender (p = 0.21), residence (p = 0.28), education (p = 0.61), occupation (p = 0.44), location of lesions (p = 0.283) and duration of lesions (p = 0.77). Significant difference in stigma score was observed for type of skin lesions (p = 0.03), marital status (p = 0.01) and age group (p = 0.04). Patients with polymorphic skin lesions reported to have slightly higher stigma (12) as compared to patients with monomorphic lesions (10.5). EMIC stigma score and its relation with clinico-demographic parameters are shown in **Table 4**.

**Table 4:** Clinico-demographic characteristics of patients in relation to stigma score (n = 120).

Characteristics	n (%)	Median	p value
**Gender**			
Male	76(63.3)	11	0.216
Female	44(36.7)	12	
**Age**(Years)			
18–30	75(62.5)	12	0.040
31–50	41(34.2)	9	
+51	4(3.3)	8	
**Marital status**			
Married	79(65.8)	10	0.019
Unmarried	41(34.2)	12	
**Residence**			
Urban	11(9.2)	11	0.282
Rural	109(90.8)	11	
**Education**			
Illiterate	40(33.3)	10	0.614
Primary school	17(14.2)	13	
Secondary school	32(26.7)	11	
Intermediate	21(17.5)	11	
Graduate/more	10(8.3)	12.5	
**Occupation**			
Farmer	14(11.7)	10.5	0.446
House wife	28(23.3)	10	
Business	8(6.7)	11	
Student	13(10.8)	13	
Labor	39(32.5)	11	
Unemployed	11(9.2)	12	
Job	7(5.8)	11	
**Lesions location**			
Exposed parts	31(25.8)	12	0.283
Unexposed parts	2(1.7)	6	
Both	87(72.5)	11	
**Skin lesions**			
Monomorphic	56(46.7)	10.5	0.035
Polymorphic	64(53.3)	12	
**Disease duration**(Year)			
<1	46(38.3)	11	0.771
1–5	42(35)	12	
>5	32(26.7)	11	

n = total number of patients

On multiple regression analysis type of skin lesions, distance to PHC shown statistically significant association with the patient delay (p<0.05). Gender and distance to PHC had a statistically significant association with health system delay (p<0.05). Similarly, gender, distance to PHC as well as type of skin lesion was also found to have significant association with total delay (p<0.05). The details of various determinants associated with delay in seeking treatment are depicted in **Table 5**.

**Table 5:** Determinants of patient, health system and total delays among PKDL Patients (n = 120).

Variables	Patient delay			System delay			Total delay		
	β	t	p value	β	t	p value	β	t	p value
(constant)		1.948	.054		3.182	.002	3.255	.001	
Gender	-.104	-.974	.332	-.227	-2.167	**.032**	-.206	-2.007	**.047**
Marital status	-.053	-.523	.602	-.006	-.058	.954	-.034	-.350	.727
Education	-.112	-1.086	.280	-.090	-.893	.374	-.122	-1.234	.220
Occupation	.003	.029	.977	-.044	-.402	.688	-.027	-.248	.805
Residence	-.063	-.692	.491	-.176	-1.974	.051	-.149	-1.712	.090
Skin lesion type	-.205	-2.144	**.034**	-.133	-1.412	.161	-.203	-2.205	**.029**
Distance to PHC	.261	2.672	**.009**	.234	2.435	**.016**	.300	3.193	**.002**

## Discussion

Visceral leishmaniasis is a disease that persists in India despite WHO's 2020 elimination target growing ever closer [20]. The National Vector Borne Disease Control Programme also reported rise in PDKL cases. Therefore, PKDL cases need to be given priority and it may pose an important challenge to VL elimination. Hence, it is timely for policy managers to revisit the strategies and adopt modification if required. We broadly examined the healthcare seeking behaviour of PKDL patients in Bihar. Much longer delay (median: 285 days) was observed for making disease confirmed as PKDL, which, indicates poor healthcare seeking behaviour of PKDL patients. Similar to our results longer patient delay was observed in other study on PKDL, -conducted in Bangladesh[6]. Longer delay was also reported in breast cancer[21] and TB [22,23]. There is currently no accurate information/data on burden of PKDL available and existing programme is not in position to either detect or manage PKDL cases at periphery level with the limited resources available to them. However, immediate diagnosis and treatment of VL and PKDL cases remains an important part of effective Kala-azar elimination programme [24].

Apart from skin changes PKDL patients does not suffer from any illness or physical discomfort. They seek treatment only when the lesions intensify or patients are of marriageable age. Furthermore, despite of kala-azar in the past, they were not aware about the linkage between kala-azar and PKDL. Therefore, effective counselling to the patients or their family members during kala-azar episode is essential. Patients working as laborers or farmers reported longer delay in seeking treatment than the patients involved with other occupation (p<0.05). Similarly, Illiterate patients reported longer patient delay as compared to the educated patients (p<0.05). Similar observation has also been documented among PKDL patients in Bangladesh [6]. The possible reasons for that may be educated people remain conscious about their health in contrast to illiterate people. Male patients were also found to have a longer delay than female patients. The possible reason may be due to the fact that females are more conscious about their personal appearance and beauty than males. Significant difference in patient and health system delay was observed based on their choices of first health care provider. Patients who approached the PHC had shortened the delay in seeking care. However, a considerable number of patients in our study initially approached to quacks, homeopathic and ayurvedic practitioners which could possibly lead to system delay. The possible explanation may be trained health workers available at the PHC. They suspect PKDL individual at the screening itself and therefore, refer the suspected individual immediately to the nearest available higher centre. Further it has also been found that longer the distance from PHC longer the delay in seeking treatment. Patients with monomorphic skin lesions showed more delay, when compared to polymorphic skin lesions in seeking care. Patients with polymorphic lesions experienced more visual disfigurement or deformities than monomorphic lesions. This may be a reason for the long delay in patients with monomorphic lesions. Furthermore, lesions initially appears as a single or few purely macular and remain localized which gradually progresses to more severe polymorphic form and spreads on whole body. Patients often confuse it with pityriasis and neglect it. Longer delay recorded under the present study may be due to poor KAP status of PKDL patients. Majority of the patients (68.3%) had poor knowledge about the PKDL. A study conducted in Bangladesh showed that 93% patients were not aware of PKDL [6]. However, a considerable number of patients (72.5%) in our study heard of kala-azar. This value is higher than the study conducted on CL in South Ethiopia (67.6%) [25] but, lower than the study conducted on VL in north west Ethiopia (87.6%) [7]. When the respondents were asked about the vector of the disease only 32.5% patients replied correctly. This value is lesser than the study in South Ethiopia 49%[25] but higher than a study conducted on CL in Pakistan 27.6% [8]. Regarding cure of the disease maximum number of respondents (78%) showed positive attitude. This might be due to the fact that almost all the patients approaching to our hospital had been referred by dermatologist or PHC. A study in Bangladesh only 21% PKDL patients had positive attitude towards the outcome of the disease [6]. However, another study in Pakistan in CL, 70.4% of the respondents had positive attitude towards the disease outcome [8]. Around half of the respondents were not aware that insecticide such as DDT/synthetic pyrethroids spraying can also control sandflies. Hence, patients as well as their families need to be informed about it. This may probably encourage other members of the community to have their home sprayed. Around half of the patients (50%) had sleeping habits in outdoors, this is slightly higher than the study conducted on CL in South Ethiopia where 40.8% respondents reported sleeping outside [25]. Hence, they are susceptible to bite of sandflies. This may be due to rural residents, lack of electric supply in rural area and poor socio economic status of the patients.

The median score of stigma found was 11, which is concordant to the study conducted on leprosy in western Nepal[26], but lesser than the score obtained in onchocercal skin disease [27]. There was a significant difference in stigma between single and married patients and among patients with different age group (p<0.05). Consistent results were also documented in other study[28]. The possible explanations may be younger age group perceive that the disease may cause problems of marriage, they may also face challenge to participate in social events, opposite sex may think less of them and more conscious about higher self-esteem.

Patients with polymorphic skin lesions under the study were more stigmatized than monomorphic lesions (p<0.05). The possible reasons may be the fact that polymorphic lesions are more awkward and look contagious to others. It may affect the self-esteem/self-image, aesthetic appearance of the affected individual. Patients who had lesions on exposed body parts had more stigma than on unexposed body parts, however, it was statistically insignificant. Median stigma score of women was 12, this value was slightly higher than men, and however, the difference was not statistically significant. Similar observation was also found in other neglected disease such as onchocercal skin disease [27].

## Limitations

This was a hospital based single centre study which may limit the generalizability. We have assessed only perceived stigma while other two types of stigma was not assessed. Therefore, results of this study should be interpreted accordingly. Patient recall bias was the other limitation for this study. Due to the long delay in approaching to our centre, patients may face difficulty to recall things regarding the appearance of first PKDL symptoms or when did the patients first approach the health care provider. Other limitations are that, we did not assess the effect of knowledge and stigma on health seeking behaviour of the patients. These factors are known to have association with the delay in seeking care [28, 29].

## Conclusions

Unacceptable high level of treatment delay has been observed. Our study revealed that a large proportion of patients have poor knowledge regarding the disease, vector and its transmission, which has effect on their attitude and practices. PKDL patients have also been stigmatized. Therefore, there is need to strengthen public awareness efforts against PKDL and related stigmatization with regard to Kala-Azar and PKDL infections. The free services for the diagnosis and treatment for VL and PKDL should be spread in the community. The study further provides the basis to National vector borne disease control program (NVBDCP) to educate the communities on clinical presentation, the need for early diagnosis, treatment adherence and curability of PKDL. Kala-azar elimination programs in India need to focus towards counselling of patients during VL episode.

## Supporting information

S1 File This is the knowledge attitude practice (KAP) and health seeking behaviour questionnaire. (DOCX)

S2 File This is the stigma questionnaire. (DOCX)
