# Supplementary material for: Knowledge, stigma, health seeking behaviour and its determinants among patients with post kalaazar dermal leishmaniasis, Bihar, India
Source: PLoS One. 2018 Sep 7;13(9):e0203407. doi: 10.1371/journal.pone.0203407 (PMC6128567; doi:10.1371/journal.pone.0203407)
Supplement: S1 File — (DOCX) [file pone.0203407.s001.docx]

Questionnaire

Date: Patient ID:

**Demographical data:**

| 1. Gender Male Female |
| --- |
| 2. Age in years : District : |
| 3. Marital status Married  Unmarried |
| 4. Education Illiterate Primary school  Secondary school Intermediate  Graduation or more |
| 5.Occupation  Farmer House wife  Business Student  Daily labour unemployed  Govt./private job |
| 6. How many people live in your household ? : |
| 7. What is your family income in a month ? : |
| 8. Residential status Urban Rural |
| 9. Effected body parts  Exposed (face, neck, limbs) Unexposed (trunk, genitals)  Exposed & unexposed |
| 10. Type of skin lesions  Macular Nodular Papular Macular & papular Nodular & papular  Mixed polymorphic |

**Knowledge towards disease and vector:**

| 11. Have you heard of kala azar? Yes No |
| --- |
| 12. Have you heard about PKDL? Yes No |
| 13. Did you ever suffer with kala azar yes No |
| 14. What are the symptoms of the PKDL?  Headache  Stomach ache  Diarrhoea  skin lesions  Fever  I don’t know |

| 15. Do you know the vector of the Kala-azar?  Sand fly  Insects  House fly  mosquito  I don’t know |
| --- |
| 16. Can you identify sand flies from common flies or mosquitoes? Yes No |
| 17. Do you know about the breeding places of sand flies?  Moist places  Cow dung  Fresh water  Crevices and cracks in house  Soil  Unhygienic conditions  I don’t know |
| 18. Do you know the preferred biting times of sand flies?  During dusk &dawn During midnight At any time During day time I don’t know |

**Attitude towards PKDL:**

| 20. Is PKDL curable? Yes No I don’t know |
| --- |
| 21. Can we controlled sandflies by Insecticide spraying (DDT/Synthetic Pyrethroids)  Yes No I don’t know |
| 22. Whether facilities at PHCs are adequate to manage PKDL cases?  Yes No I don’t know |

**Practice & health seeking about PKDL:**

| 23. First where did you go for treatment ?  Quacks  Govt. health center (PHC)  Homeopathic/ayurvedic doctors  private practitioner |
| --- |
| 24. After how many days of PKDL symptoms you sought the medical attention |
| 25. Where is the disease first diagnosed ? |
| 26. Do you use mosquito bed net while sleeping ? Yes No |
| 27. Do you sleep in outdoor? Yes No |
| 28. How to control the disease  Preventing sand fly breeding  using bed net  using mosquito repellents/coil  Maintaining hygine  I don’t know |
| 29. Source of information  Govt. health worker  Radio/TV/Newspaper  Posters / Leaflets  Neibhours / relatives  Govt./private doctors |

| 30. Do you know where the nearest Govt. Health facility is? Yes No |
| --- |
| 31. How much is the distance of health facility from your village ? |

Signature

THANK YOU FOR SPENDING YOUR VALUEBLE TIME
