# Supplementary material for: Knowledge, stigma, health seeking behaviour and its determinants among patients with post kalaazar dermal leishmaniasis, Bihar, India
Source: PLoS One. 2018 Sep 7;13(9):e0203407. doi: 10.1371/journal.pone.0203407 (PMC6128567; doi:10.1371/journal.pone.0203407)
Supplement: S2 File — (DOCX) [file pone.0203407.s002.docx]

EMIC STIGMA SCALE PKDL ID:

| No. | QUESTION | Yes  3 | Possibly  2 | Uncertain  1 | No  0 | Score |
| --- | --- | --- | --- | --- | --- | --- |
|  | Would you prefer to keep people from knowing about your disease? |  |  |  |  |  |
|  | Do you think less of yourself because of your disease? Has it reduced your pride or self-respect? |  |  |  |  |  |
|  | Have you ever been made to feel ashamed or embarrassed? |  |  |  |  |  |
|  | Do your neighbours, colleagues or others in your community have less respect for you because of PKDL? |  |  |  |  |  |
|  | Do you think that contact with you might have any bad effects on others around you? |  |  |  |  |  |
|  | Do you feel others have avoided you because of your disease? |  |  |  |  |  |
|  | Would some people refuse to visit your home because of this condition? |  |  |  |  |  |
|  | If they knew about it would your neighbours, colleagues or others in your community think less of your family because of PKDL? |  |  |  |  |  |
|  | Do you feel that this disease might make it difficult for you to marry? |  |  |  |  |  |
|  | Do you feel that this disease has caused problems in your marriage? |  |  |  |  |  |
|  | Have you been asked to stay away from work or social groups? |  |  |  |  |  |
|  | Have you decided on your own to stay away from work or social gatherings? |  |  |  |  |  |
|  |  |  |  | Sum |  |  |
